# Cognitive models facilitate real-time inference of latent motives

**DOI:** 10.1038/s41598-026-37587-8

**Published:** 2026-01-28

**Authors:** Anderson K. Fitch, Peter D. Kvam

**Affiliations:** 1 Department of Psychology, University of Florida, Gainesville, FL USA; 2 Department of Psychology, The Ohio State University, Columbus, OH USA

**Keywords:** Mathematics and computing, Neuroscience, Psychology, Psychology

## Abstract

The ability to continuously make inferences about another person’s latent states from their behavior is integral to how people behave in social situations, yet is lacking from most artificial intelligence (AI) systems. The present study tests the capacity of cognitive models to assess latent motives in real time by evaluating different deep neural networks trained to infer a human player’s intent during a continuous control task. These networks were trained by (a) directly using observable information or (b) selecting important features by estimating the parameters of a generative model of movement behavior inspired by approach-avoidance theory. Comparisons of classifier accuracy suggest that latent model parameters predict a participant’s intent at a level exceeding human performance. Furthermore, classifier performance was best when model-based inferences were combined with summary statistics about behavior, yielding faster and more stable network training compared to networks that had no manual feature extraction. Equipping AI with cognitive models is a promising avenue for developing explainable, accurate, and trustworthy systems.

## Introduction

Humans readily and continuously assess the attitudes, moods, and goals of those around us. While we cannot directly observe these hidden states, we use patterns of behavior to make deductions about the thoughts and motivations guiding one another’s actions. This ability comes naturally to most people [with some exceptions in conditions like autism spectrum disorders^1^], and is not limited to inferring the states of other people; even the movement of simple geometric shapes is commonly interpreted in terms of their dispositions or social motivations^2^. This type of inference, where a person makes judgments about hidden or latent states on the basis of another’s outward actions, is a critical component of Theory of Mind [ToM^3^], strategic decision-making, and social interactions more broadly^4,5^.

Artificial intelligence (AI) has come leaps and bounds in its predictive power, yet its ability to explain behavior in a theoretically meaningful way has not progressed at the same pace. Complex AI systems, like most deep neural networks, are considered “black boxes” because we cannot explain *why* an AI makes certain predictions by directly examining a network’s learned weights and structure^6,7^. Likewise, human cognition might be a “black box” for an AI system because behavior is rarely, if ever, a direct measurement of a cognitive process. This mutual lack of transparency – between a person and an AI system – may undermine a user’s trust and hamper an AI’s ability to make good predictions about a person’s behavior^8,9^. This becomes dangerous in many applications of AI systems, such as when they are asked to interact with human decision makers in challenging social environments like driving – where inferring other drivers’ latent motives related to merging, turning, passing, and other actions are critical to functioning successfully^10,11^.

One solution to this problem may come from existing tools in the psychological and cognitive science literature. Cognitive models that quantify latent traits and processes from observed outcomes^12–14^ help psychologists understand and predict human behavior by connecting noisy, difficult-to-parse behavior with stable, meaningful model parameters. Consider the assessment of two oncologists. One oncologist diagnoses frequently, leading to more hits and fewer misses, but also many false alarms, where patients undergo unnecessary treatment. The other oncologist is more conservative, only diagnosing when they feel confident, resulting in fewer false alarms, but occasionally someone is missed. Without a model, a patient or hiring manager may only compare numbers, trading off hits, false alarms, misses, and correct rejections. But with a model like signal detection theory^15^, behavior can be quantified in terms of sensitivity/discriminability and response biases/criterion. This allows a person to more directly assess a doctor’s ability and their level of caution. These parameters, which represent latent processes and traits, may instill some degree of human-informed ToM in AI systems. There are a variety of cognitive models in widespread use across domains like preferential and perceptual choice, perception, memory, and other tasks^16–18^ – allowing for inferences about many different latent processes.

However, many cognitive models are currently limited by the speed at which they can be fit and applied to data, making it difficult to embed within AI systems that must make inferences in real time. This paper aims to bridge this limitation by creating AI systems that can effectively use latent information extracted by cognitive models to make real-time inferences about a person’s latent motives. To do so, we first examine how humans and AI diverge in their approach to social or latent inference tasks. Next, we introduce cognitive modeling in general as a solution to bridge the two methods of latent intent inference. We then present a task, model, and modeling approach that enable real-time intent inference to demonstrate how deep neural networks can use cognitive modeling to make fast, interpretable, and reliable inferences about the latent motives driving human behavior.

### Artificial intelligence and theory of mind

Despite the importance of latent states in human interactions, most AI systems tend to make inferences over observable outcomes rather than making inferences or attributions about underlying motives^19^, though simple agents that possess some ToM are in development^20^. By leveraging large data sets, complex architectures, and modern computing power for extensive training, AI models can outperform people in many domains^21^. Over the last few decades, two-player games like chess^22^ and variants of poker^23^ have seen AI systems outperform the best human players. Even social games with many players, like 6-person poker, have seen AI systems outperform human experts^24^. Performance in these more complex games is particularly striking because unlike two-person competitive zero-sum games where an AI system could plausibly approximate some Nash equilibrium from game theory, non-zero-sum games do not have a known polynomial-time algorithm to solve them^24,25^. Despite an inability to explicitly define a tractable, optimal strategy, people and AI systems can learn complex social games, though often in very different ways. For example, AlphaGo Zero discovered numerous strategies in the game Go from self-play – notably using a strategy typically taught to beginners very late into training^26^. In cases like these, performance might be better when learning from only self-play and not human experts as was implemented in an earlier model^27^, but in tasks that are inherently about predicting human behavior rather than achieving a particular state of the world, learning from humans may be necessary to accurately predict a person’s behavior^21^.

Machine ToM, and in particular deep learning-based approaches, are commonly tested through false belief tasks^28^. Some approaches to machine ToM focus on network structure to learn the relationship between observed behavior and latent motives or beliefs, such as representing traits, states, and current predictions in three different networks^29^. In contrast, others approach machine ToM by specifying an explicit model of human behavior^30^. Even large language models have been applied to verbal false belief tasks^31–33^. In addition to verbal false-belief tasks, others have used discrete-state and discrete-time grid worlds with observers or agents themselves that require inference of hidden states / information to test ToM abilities^29,34,35^.

Despite the progress on particular tasks, there is a pressing need to extend the scope of ToM-like abilities to include tasks involving strategy identification, ranging from human-machine teaming to cybersecurity, self-driving cars, and air traffic control^36–39^. For example, a random forest reinforcement learning algorithm was able to classify participants’ search and choice strategies^40^. The AI performed best when given input data in the form of selected features, rather than raw mouse data. This suggests that, while a cognitive model might not be necessary to do strategy identification, some amount of user-defined feature selection was beneficial. Indeed, intelligent feature selection is key to accomplishing many human-like capacities, as illustrated in transformer models^41^. Identifying key interpretable features may therefore unlock the key to understanding and representing the contents of the human mind during strategic decision and control tasks.

### Cognitive models and continuity

Using a cognitive model as the front-end for theory of mind resembles feature selection, but instead of providing summary information about an observable phenomenon, it quantifies features (i.e., parameters) of a latent process. These parameters can provide both trust and explainability, as they correspond to processes that are easy to articulate and communicate^42^. For example, the model CogToM uses a cognitive model of instance-based learning theory (IBL) to predict the actions and false beliefs of reinforcement learning agents in a grid-world^34^. The IBL observer had performance similar to that of human participants observing the behavior. Importantly, the IBL observer made the same types of mistakes as participants. This suggests that cognitive models might be leveraged to make AI think *like* a human.

There are two critical limitations to this approach. First, simple grid worlds and discrete-time tasks make it possible to take shortcuts or heuristics that approximate what an agent with theory of mind might do without actually leveraging meaningful inferences about latent states^43,44^. Second, operating in the real world involves grappling with tasks in continuous (real) time and continuous space^45–48^. As a result, the grid-world types of scenarios and experiments that have been done so far^29,34^ can only shed so much light on intent inference and theory of mind in real environments.

The latter issue of real-time inference presents a serious hurdle for AI systems as well as for cognitive models. Many generative cognitive models of continuous behavior lack an explicit likelihood function to obtain the posterior probability or likelihood of a set of model parameters given some data, making them prohibitively slow to fit in real time using traditional methods^49–51^. However, recent advances have made simulation-based methods more accessible, like using neural networks that learn from simulated data to estimate cognitive model parameters^51–54^. Neural-network based approaches amortize the inference process, splitting up the computational load of data simulation and parameter estimation. This front-loading means that trained networks can be used to estimate the parameters of most cognitive models in real time.

Put together, we suggest that AI can be augmented with ToM capabilities by equipping them with cognitive models to extract latent features in real time using pre-trained deep neural networks. This provides the first steps toward social AI, enabling inferences about latent processes and states on the basis of observed behavior. If these models are a useful approximation of the underlying system^55^, then their parameters provide information about that which is hidden in the observable environment by inverting the mental processes that give rise to behavior, rather than simply predicting future behavior from past behavior^56^, overcoming current barriers for machine ToM. In this paper we will demonstrate how cognitive model parameters can be used in AI systems to improve accuracy when classifying participants’ latent motives. These types of inferences are critical for effective machine ToM, but in order to realize these benefits, cognitive models must be fit in real time. In the next section, we examine a continuous-time, continuous-space task that provides a testbed for constructing and validating these real-time approaches to intent inference.

## Methods

To explore how AI can use behavior to make inferences about latent motives, we first created a task where human decision-makers had a (hidden) goal to continuously pursue. This experiment featured a novel continuous control task where pairs of human participants and computer opponents each moved a “ship” around the screen and earned points according to how well they achieved their assigned objectives. A total of 79 participants were recruited through department listservs. Participants were between 18 and 22 years old ($$M = 19.38, SD = 1.26)$$ and 87% identified as female ($$n = 69$$), 6% as male ($$n = 5$$), 4% as non-binary ($$n = 3$$), and 3% preferred not to answer ($$n = 2$$). Sessions lasted approximately an hour and participants were compensated a base payment of $15 with a bonus between $0-$10 based on points earned during the task. Compensation was delivered via a Visa Prepaid debit card.

Critically, we do not directly examine behavior on this task. Instead, we used data from the top performers on this task to create videos to show a second set of participants, whose task it was to identify what goal the human player (recorded in the video) had been assigned. This allowed us to have a ground truth, as the experimenters knew for each video what goal the human player had been assigned. We can compare this to the inference made by independent human judges and deep learning systems that were trained to determine latent motives. Next, we provide a short description of the task that was shown in the videos, followed by the paradigm that our focal participants followed to evaluate the videos.

### Continuous control task - recording

**Figure 1:**
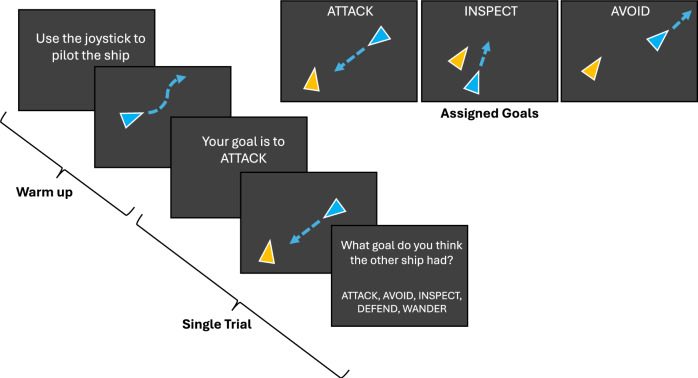
Continuous control task diagram. On each 10-s trial, a participant was assigned a goal and competed with a computer opponent with an independent goal carried out by different movement profiles.

Participants in the recorded videos played a continuous control task programmed in MATLAB, outlined in Fig. 1. This task was presented as a ship piloting game, where the participant controls the movement of a triangle with a joystick and interacts with a computer opponent. In each video, the human player had been tasked with moving the ship around the screen to earn points based on their goal, while the computer opponent moved with one of five movement profiles.

Specifically, in each of the videos, the human player had been assigned one of three goals: *Attack* (collide with the other player), *Avoid* (stay away from the other player), or *Inspect* (stay near but do not collide with the other player). The opposing ship, controlled by a computer, had been assigned one of five corresponding profiles: *Aggressive* where it moves towards the participant (mirroring *Attack*), *Shy* where it moves away from the participant (mirroring *Avoid*), *Curious* where it stays near the participant (mirroring *Inspect*), *Defensive* where it stays between the participant and a location unknown by the participant, or *Wandering* where it wanders around the screen, only avoiding edges and collisions. The code to produce computer movement is available on https://osf.io/cftuy/. Participants earned points by accomplishing their goal on each trial and lost points for unwanted collisions. The pairings of the participant’s goal and opponent’s movement profile naturally produced some trials where movements between the ships conflicted, and others where movements were mutually beneficial. For example, the pairing of *Attack*-*Aggressive* or *Inspect*-*Curious* was a considerably easier trial than *Attack*-*Shy* or *Inspect*-*Aggressive*.

The three participant goals were crossed with the five computer movement profiles, for a combination of 15 different goal-profile condition pairings that were repeated 6 times each, for a total of 90 trials. Participants were randomly prompted on half of the trials to infer the goal of the computer opponent. While the computer opponent was programmed with behavioral profiles, rather than having latent motives or goals, these profiles (*Aggressive, Shy, Curious, Defensive, Wandering*) approximated five goals: Attack, Avoid, Inspect, Defend, and Wander, respectively. This data will not be included here because inferences of computer behavior by a player are not inferences of human intent, but rather reflect a person’s mental model of the computer.

To directly compare assessments of human observers and machine-based systems, we took data from the eight participants who earned the best total score during the continuous control task, and used these to create videos from each combination of conditions. This ensured that videos featured behavior from participants who were, on average, successful in the task.

### Human benchmark - viewers

To assess machine inference compared to human inference, we had people watch videos of behavior in the continuous control task outlined in the previous section. The goal of the human participants was to infer the player’s assigned goal based on a short clip of behavior from the control task. On each trial, participants had to choose whether they thought the player’s goal was to *Attack*, *Avoid*, or *Inspect* the computer opponent.

In addition to a human benchmark for overall accuracy, we also aim to assess how intent inference unfolds across time. In other words, do people tend to have different expectations one second into the trial and at the end of the trial? It might be reasonable to expect that *Attack* and *Inspect* goals might be difficult to delineate after only one second of movement because they both move toward the opponent when far away. To test this, we compared goal inference after watching 1 second, 4 seconds, 7 seconds, and 10 seconds of a trial. Given that there are three participant goals, five computer behavioral profiles, and four duration conditions, we assess a total of 60 videos for each goal-opponent-duration pairing in a random order, repeated twice for a total of 120 trials per participant.

Different groups of people may have differences in ToM abilities, with deficits commonly observed in people with autism spectrum disorder (ASD)^1,4,57,58^. However, there is also some evidence that adults with ASD might play more video games than a typically developing control group^59^. The game-like nature of the continuous control task may allow participants with ASD, who might otherwise have deficits in ToM, to make goal-inference judgments accurately. It is also possible that monetary incentivization may influence accuracy. To test these possibilities, we recruited three different samples to establish a human benchmark: an unpaid convenience sample recruited through SONA (compensated with undergraduate class credit), a paid general population sample recruited through Prolific, and a paid Prolific sample who report being diagnosed with ASD.

For all samples, the experiment was delivered online through Qualtrics. Videos were embedded in Qualtrics using JavaScript. A total of 97 people completed the experiment from the SONA sample. These participants were between 18 and 35 years old ($M = 19.98$, $SD = 2.32$) and 87% identified as female ($n = 84$), 12% as male ($n = 12$), 1% as non-binary ($n = 1$). Sessions lasted approximately half an hour and participants were compensated with class credit. For both Prolific samples, participants were compensated at an hourly rate. A total of 84 people completed the experiment from the Prolific general population sample. These participants were between 19 and 69 years old ($M = 37.31$, $SD = 12.23$) and 45% identified as female ($n = 38$), 50% as male ($n = 42$), 5% as non-binary ($n = 4$). Lastly, a total of 100 people completed the experiment who had been pre-screened for autism spectrum disorders [ASD] through Prolific, yielding the Prolific ASD sample. These participants were between 19 and 64 years old ($M = 34.86$, $SD = 9.86$) and 30% identified as female ($n = 30$), 57% as male ($n = 57$), 12% as non-binary ($n = 12$), and one person preferred not to answer. An analysis of variance (ANOVA) on accuracy with sample group as a fixed factor and participants as a random factor suggests that the different groups did not differ meaningfully in terms of performance ($BF_{10} = 0.004$), so we group all the human participants together in the real-time accuracy results we report below.

### Approach avoidance

To develop a useful cognitive model of behavior in the continuous control task, we began with approach-avoidance theory^60,61^. Approach-avoidance theory suggests that behavior is a product of the conflict between the benefits of drawing closer to something and the harms of getting too close. For example, approaching a campfire provides warmth and light, but getting too close results in a burn. Classically, approach-avoidance tensions are modeled as the difference between two monotonic gradients. Both the approach and avoidance gradients rise as the distance (real or psychological) to the target decreases – corresponding to stronger motivation or urgency to interact with another player as they get closer. Avoidance gradients tend to have a steeper slope than approach gradients, resulting in approaching behavior from a far distance and avoiding behavior from a close distance. In other words, when far away, the balance of approach-avoidance gradient activation favors approaching. As you draw closer, that balance shifts towards greater relative avoidance activation until avoidance is greater than approach. This results in distance maintenance behavior focused at a single point where the two gradients intersect, often producing oscillatory or asymptotic movement behaviors around that favored point^61^.

### Global-local objective pursuit (GLOP) model

**Figure 2:**
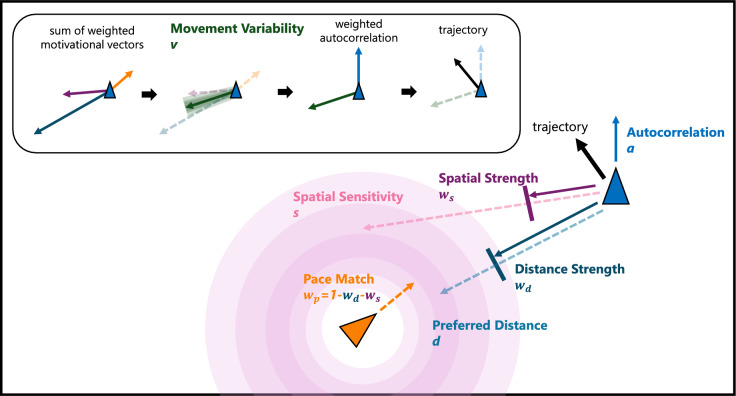
Global-local objective pursuit (GLOP) model diagram. Intended movement, represented by the motivational vector, is determined by the weighted sum of the preferred distance, spatial sensitivity, and pace matching vectors plus movement variability. The trajectory vector, which represents the next step of movement, is a weighted sum of the motivational vector and the autocorrelation vector.

The global-local objective pursuit model, illustrated in Fig. 2, extends approach-avoidance theory by focusing on the point where approach-avoidance gradients intersect as an “objective” in participant movements. Changes in movement are driven in part by the motivation to increase (avoid) or decrease (approach) the distance between the self and the opponent. However, this model extends approach-avoidance by having multiple goals, including motivation to maintain a particular distance (preferred distance), maintain a good position in space (spatial sensitivity), and maintain a particular trajectory relative to the other player (pace matching). This creates an integrated theory of local objectives (describing how the two players influence one another) and global objectives (describing desirable and undesirable locations or states of the world) – hence the global-local objective pursuit title.

Formally, the average trajectory a person takes in this model is determined by a weighted sum of two vectors: a motivational vector and movement autocorrelation. The motivational vector is itself a weighted sum of three vectors: preferred distance, spatial sensitivity, and pace matching vectors.

The GLOP model approximates continuous time using a sampling / updating rate of 60 Hz. At each time step, the model calculates the distance between the participant and the opponent along with a unit vector pointing from the participant to the opponent ($\hat{t}$). With this vector, we can calculate a preferred distance vector ($v_{dist}$), inspired by approach-avoidance theory (as a sample from the vector field created by the gradients), that points from the current location of the participant to the location – towards or away from the other player – that maintains their preferred distance relative to the other player. Preferred distance is a free parameter (*d*) that varies from 0 to 1, scaled by the maximum possible distance in the task ($dist_{max}$; from one corner of the screen to the other). The greater the discrepancy between current distance and preferred distance, the stronger the pressure will be to move to maintain the preferred distance, and thus the movement vector based on preferred distance will have a greater magnitude – resulting in faster movement. Formally, the preferred distance vector is calculated as

1 $$\begin{aligned} v_{dist} = [\sqrt{(p_x-o_x)^2+(p_y-o_y)^2} - (d \cdot dist_{max})] \cdot \hat{t} \end{aligned}$$

where $\textbf{p} = [p_x, p_y]$ is a participant’s current position and $\textbf{o} = [o_x, o_y]$ is the opponent’s position.

The spatial sensitivity vector ($v_{spatial}$) represents the strength of a global goal, which is a participant’s desire to occupy a “good” location in space, irrespective of the exact vector to the other player. This creates a “global” goal – that is, preferences relative to the positions a player could occupy across the entire space (screen) as opposed to preferences that relate strictly to the other player. For example, a participant does not want to get backed into a corner when trying to avoid the other player; therefore, their global preferences may involve escaping the corner of the screen to go somewhere else, even if their route to escape runs near or through the opponent. Naturally, this goal can conflict with local goals – escaping a corner may require a participant to go nearer to the other player than they would like en route to a more desirable position on the opposing side.

Formally, $v_{spatial}$ is calculated by sampling from a $50 \times 50$ grid of locations on the screen and evaluating the goodness of each one according to the participant’s distance preferences. For each of the 2,500 points in the grid, the distance from the opponent (distance matrix) and a vector from the participant’s current location (toward matrix) are calculated. The distance matrix is then used to calculate a preference strength matrix based on preferred distance. To do this, we subtract the difference between each grid location and the player’s preferred distance (a difference of 0 would be a grid location exactly at the preferred distance) from the maximum distance, and divide by the maximum distance. This gives a matrix $\textbf{D}$ of normalized values, where 1 is a most-preferred location and 0 is a least-preferred location. This preference strength matrix is then taken to the power of *s*, where *s* is the spatial sensitivity parameter. Values of $s > 1$ result in a steep preference gradient where behavior is driven by only a few specific locations the player would ideally like to be. Conversely, values of $s < 1$ result in a shallower gradient where the player is influenced by good but suboptimal locations but strongly avoids the very worst locations. The spatial sensitivity vector $v_{spatial}$ is the resulting preference strength matrix modulated by a participant’s sensitivity to these preferences or activations. Formally, it is calculated as

2 $$\begin{aligned} v_{spatial} = \sum _i \sum _j \textbf{D}_{i,j}^s \cdot [p_x-i, p_y-j] \end{aligned}$$

where $\textbf{p} = [p_x, p_y]$ is a participant’s current position on the screen and $\textbf{D}_{i,j}$ is the matrix quantifying the attractiveness of all the sample points on the screen.

Finally, the pace matching vector ($v_{pace}$) describes the direction and speed a participant wants to take relative to the direction and speed of the opponent. This vector allows the model to incorporate not only where the opponent is, but where a participant expects the opponent will be. For example, a participant who is attempting to intercept the other player might not want to simply head to where the opponent is – undoubtedly falling behind – but rather aim to cut them off at wherever they are headed. Therefore, they should move both toward the opponent and in the direction they expect the opponent to go at any moment in time to intercept them^62^. More generally, a participant’s movement should be determined not just by the arrangement of players on the screen but by their dynamic expectations about the future locations of the players – reflected in the relatively simple pace-matching component of the model.

The overall direction that a participant is motivated to go is represented as a weighted average of the preferred distance, spatial sensitivity, and pace match vectors, called the motivational vector ($v_{motivation}$). The relative weight of the preferred distance vector in the motivational vector is given by the free parameter distance strength ($w_d$) and the relative weight of the spatial sensitivity vector is given by the free parameter $w_s$, while the weight of the pace matching vector is $1-w_d-w_s$. Thus, the motivational vector is given by the equation:

3 $$\begin{aligned} v_{motiv} = (v_{dist} \cdot w_{d}) + (v_{spatial} \cdot w_{s}) + [v_{pace} \cdot (1-w_{d}-w_{s})] \end{aligned}$$

There may be some uncertainty or imprecision in a participant’s motivational vector, added to the model through a freely estimated movement variability parameter *v*. Movement variability naturally increases as the magnitude of the preferred distance, spatial sensitivity, and pace match vectors increase, scaled by *v*. That is, it is harder for someone to control their precise movement when they are going faster. With movement variability added to the motivational vector, the trajectory of the next step is then calculated as a weighted average of an autocorrelation vector and the motivational vector, called the trajectory vector. Autocorrelation with the previous step allows for the smooth patterns of movement people typically exhibit. This smooth movement behavior is a result of natural limitations on how quickly humans can perceive and respond to changes in their opponent’s behavior using the joystick. Like before, the relative weight of the autocorrelation is given by the free parameter *a*. Thus, the trajectory vector is given by the equation:

4 $$\begin{aligned} v_{traj} = (v_{autocorr} \cdot a) + [(v_{motivation} + v_{variability}) \cdot (1-a)] \end{aligned}$$

By balancing local and global objectives through competing motivational tensions, the GLOP model can produce aggressive, defensive, or curious behavior in continuous time and space through sensitivity to relative positions in space between two agents, and maintenance of a good position relative to external boundaries. While we explore the movement of two agents in this paper, the GLOP model can be extended to respond to an arbitrary number of moving agents or stationary objectives.

### Model fitting

To fit this model in real time, we trained a neural network to approximate the relationship between data and model parameters, using the cognitive model to create synthetic data on which the network could be trained. To accomplish this, a large amount of data was simulated from the model, varying its parameters related to pace/distance matching, spatial sensitivity, autocorrelation, and movement variability. To enable real-time model fitting, the end of each simulated trial was censored so that a limited amount of data from each trial was fed into the neural network for each input. This allowed the network to produce an output for any length of input, and therefore can be iteratively fit during a sequence of behavior to continuously update its outputs. The resulting sets of simulated data were then used as inputs to a neural network, with the parameters used to generate each data as the output. The neural network was then trained to approximate the relationship between data and parameters, using an ADAM supervised learning algorithm^53,63^.

The first step in this process is data simulation. In the experiment, data is generated by behavior in pursuit of a goal matched with the five different movement profiles of the computer opponent. While we can make some predictions about the relationship between model parameters and goals, like that preferred distance is expected to be highest during *Avoid*, followed by *Inspect*, and lowest for *Attack*, parameter estimates are expected to have relatively high variability within a goal condition, due to individual differences between participants. Therefore, simulated data was not binned into predicted goal-profiles. Rather, data was simulated from a set of parameters that did not necessarily correspond to a specific goal.

A total of 100,000 simulated trials were generated from the model. Parameter distributions used to generate simulated data, which served as priors in a Bayesian framework^52^, are described in Table 1. For the motivational weights that must sum to one ($w_s, w_d, w_p$), we drew each weight from an independent Gamma distribution and divided each weight by the sum of the weights. Because the computer opponent had five different movement profiles or conditions, we simulated 20,000 datasets for each movement profile. When developing the model and tuning parameter distributions, simulated trajectories were plotted to visually verify that the model was producing reasonable movement in the task.

**Table 1:** Prior parameter distributions for simulated data from the GLOP model.

Parameter	Distribution	Parameterization
*d*	Beta	(.3, .5)
$w_d$*	Gamma	(2, 2)
$w_s$*	Gamma	(2, 2)
$w_p$*	Gamma	(2, 2)
*a*	Beta	(5, 1)
*s*	Gamma	(2, 2)
*v*	Gamma	(2, 5)

*Weighting parameters were drawn independently from Gamma distributions before normalizing the sum to one.

After simulating data with known parameter values, we first removed trials where a collision occurred on the first time step. Next, we censored the length of each trial with an independent random draw between two and the maximum number of time steps that occurred in the empirical data. If the length of the trial was shorter than the randomly drawn censor length, the trial was left unchanged. Finally, we used supervised learning to train a recurrent neural network on the relationships between player movements and model parameters. To ensure the predictions were constrained to each parameter’s theoretical range, we logit-transformed parameters that were bounded on both sides and log-transformed parameters that were bounded at 0.

To assess the performance of our parameter estimation network, we randomly held out 20% of the data for a validation set. This subset of the data did not influence network weights during training and therefore allows us to test the degree of overfitting (validation accuracy decreasing across iterations) in our network. To test the identifiability of our parameter estimation network, we simulated another 20,000 trials as outlined above for a test set. Lastly, the model was validated using fits to empirical data to generate posterior predictions.

### Parameter estimation network

The recurrent parameter estimation network is structured as follows: A sequence input layer accepts a $5 \times 138$ sequence of simulated input data. The first column contains summary information, including the opponent’s movement profile, the mean and variance of the angle between players, and the mean and variance of approaching/avoiding movement (1 = always approaching, -1 = always avoiding). This was followed by 5 rows of time-series data: a flag (0 or 1) identifying if the players are still moving (e.g., have not yet collided), the x coordinate of the participant, the y coordinate of the participant, and the x and y coordinates of the computer opponent.

These input data feed into a gated recurrent unit (GRU) layer. This layer is critical to recurrent networks, as the GRU layer provides a sort of memory for the network. The present GRU layer contains as many units as the input layer is long, meaning that it can use everything that has happened throughout the entirety of the time series to inform its evaluation of the final state of the system (end of the trial). The GRU layer is followed by 3 fully-connected layers with swish activation functions on each layer of nodes. The first fully connected layer contained 150 nodes, followed by 50 nodes, and finally 15 nodes. After the 3rd swish activation function transformation, a fully-connected layer containing 5 nodes for the 5 model parameters fed into a regression layer that predicted the parameters used to generate the simulated input data.

We include a dropout layer after the first swish layer with a 0.5 probability of randomly knocking out each node in that layer to measure uncertainty regarding parameter estimates^51^. This random dropout layer gives the network slightly different predictions when sampled from, where each sample represents a draw from an approximate Gaussian process, forming a joint posterior distribution over the parameter estimates^64^.

This network was trained using the ADAM optimizer for 60 epochs with z-score normalization of inputs. While the network reached a stable performance plateau there were no signs of overfitting, so training continued for the full duration. Network architecture and hyperparameters were hand-tuned to consistently produce good parameter recovery without automatic tuning to approach optimality. Initial architecture was based off of previous work fitting cognitive models^51^.

### Classification networks

To explore how model parameters influence intent classification, we trained five different networks in addition to the parameter estimation network. These classification networks were designed to categorize a set of inputs based on which goal a participant had been assigned. Their objective was to take a set of inputs – some combination of raw behavior position data, estimates from the parameter estimation network, and summary statistics about behavior on that trial – and map them onto the latent motive or goal the human player had on each trial. Empirical data were replicated and censored to every length shorter than the observed trial to permit real-time intent inference. Although the parameter estimation network used to fit the model outlined above was trained on simulated data and fit to empirical data, classification networks were trained and tested on empirical data. Because classification networks must be trained on empirical data, the data from 79 participants was split into three sets. First, the test set consisted of all the trials from the eight participants used to generate videos for the human benchmark; this ensures that the machine test set contains all trials judged by humans. Of the remaining 71 participants, 8 were randomly selected to serve as the validation set, with the remaining 63 participants serving as the training set. This naturally means that the test set features the players that earned the most average points from successfully accomplishing their goal, thus we might infer that there is greater noise in the training and validation sets. This suggests that networks might then perform interpolation, which is generally preferable to extrapolation when using feed-forward networks^51,65^. Ultimately, this approach allowed for human participants to judge high-quality data without necessitating hand-selection of trials.

The first classification network received model parameter estimates (mean and variance of 100 samples from the estimation network) from a participant’s data, as generated by the parameter estimation network described above, and learned to predict a participant’s goal from their model parameter estimates. The second network received the raw positions of the participant and opponent with a length flag as inputs. The third network received summary statistics as inputs, like the first column of the parameter estimation network. The fourth network was an ensemble that received the predictions made by the position and model-parameter networks, allowing it to use both raw behavior and model-based inferences about that behavior. Finally, the last ensemble network received predictions from the summary-statistic and model-parameter networks as inputs. Together, the performance of these classifiers will take the first steps to understanding how model parameters can predict latent motives, and how that type of classification compares to raw data (position) and summary statistics.

To ensure that network structure did not bias classification accuracy, each network was structured in a nearly identical way and trained for 4685 iterations (time-series) or epochs (features). All networks started with a sequence input layer, structured according to the type of data input to the network. The position network accepted time-series data requiring a GRU layer, while this layer could be omitted from networks accepting features rather than time-series data (Model, Summary, Ensembles). These layers were followed by a series of fully connected layers separated by swish activation functions. Fully connected layers had 100, 60, 30, 20, 10, and 3 (number of classes to predict) nodes each. The final fully connected layer was followed by a softmax layer to convert the output into probabilities that sum to 1. A Dropout layer was added to the first fully connected layer to prevent overfitting. Like the parameter estimation network, architecture and hyperparameters were hand-tuned such that all networks trained consistently and did not suffer from overfitting without tuning for optimality to maintain consistency across networks for comparisons. Data, materials, model code, and trained networks are available on https://osf.io/cftuy/.

## Results

To investigate how AI intent inference may benefit from estimates of latent cognitive processes through modeling, we first examined how well we can recover our model and posterior prediction error across conditions. Second, we compared classifiers to understand the extent to which model parameters alone can predict intent and whether the addition of model parameters provides the network with information above and beyond what is directly observable.

### Model performance

The first step toward using a cognitive model to assist with latent intent inference is to confirm that the model is useful in principle – that is, establishing that its parameters are meaningful and useful in quantifying and predicting behavior. To do so, we first checked that the parameters of the model could be estimated from the data that we collected. Correctly identifying parameter values from only 10 seconds of behavior is potentially a high bar – it could be the case that only one or two of the GLOP parameters are identifiable from the data. Therefore, the first step is to simulate 20,000 artificial test trials from the model and then attempt to recover (identify) the original parameters of the simulation.

#### Parameter recovery

If the model is a close approximation of the true underlying processes, the act of parameter recovery (comparing the correlation between true and predicted parameter values) on our simulated data can give us an upper benchmark on how good our parameter estimates can be.

**Figure 3:**
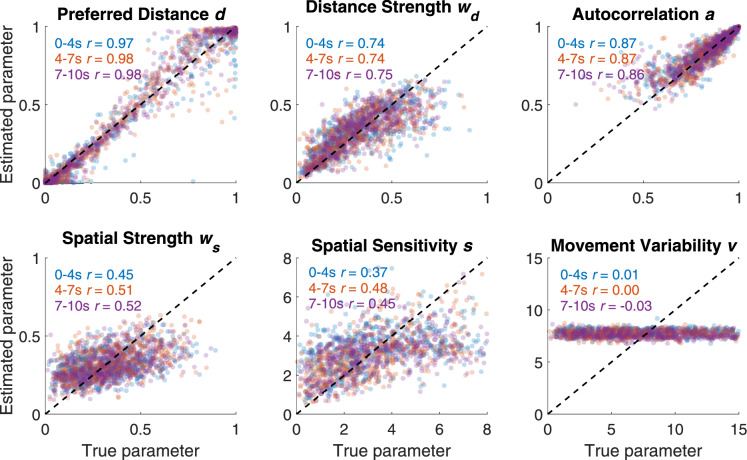
Parameter recovery. Plots show the correlation between the true parameters used to simulate data and the network’s predictions of those parameters, binned by sequence length. While preferred distance and autocorrelation showed the best recovery, all parameters except for movement variability show some recovery.

Parameter recovery for the test set was excellent for preferred distance ($r >=.97$), distance strength ($r >=.74$), and autocorrelation ($r >=.86$), suggesting that the task is highly informative for local objectives (see Fig. 3). Spatial strength ($r >=.45$) and spatial sensitivity ($r >=.37$) demonstrated less impressive but still somewhat effective parameter recovery, with both parameters showing better recovery for longer trials that were greater than four seconds ($r >=.51$ and $r >=.45$, respectively), which may indicate that global objectives are more difficult to identify from behavior than local objectives. Finally, movement variability could not be estimated, with nearly no correlation between true and predicted values ($r <=.01$). Fortunately, we have little reason to believe that movement variability, accounting for uncertainty or imprecision in movement, should have strong differences between conditions. We freely estimate movement variability despite poor identifiability to absorb unaccounted-for error that would otherwise contaminate remaining model parameters, similar to how across-trial variability can facilitate the estimation of other parameters in a diffusion model without itself being identifiable^66^. Poor recovery of a parameter typically limits its interpretation, granting little confidence when making comparisons between groups or conditions. When neural networks use parameters as inputs, training optimization naturally drives uninformative parameters to have less influence and highly informative parameters to have greater influence, thus the inclusion of poorly estimated parameters in classification networks produce, at worst, noise that can be ignored, but may provide some non-zero amount of meaningful information. Lastly, parameter recovery was similar for the training, validation, and test sets, suggesting that the network did not suffer from overfitting.

#### Posterior predictive checks

A key piece of model-based intent inference, whether embedded within AI or used for more traditional modeling applications, is having a model that actually does a good job of explaining and predicting behavior. The next step of checks aimed to establish the quality of the model as it reflects the real data by comparing its best predictions and real behavior side by side.

**Figure 4:**
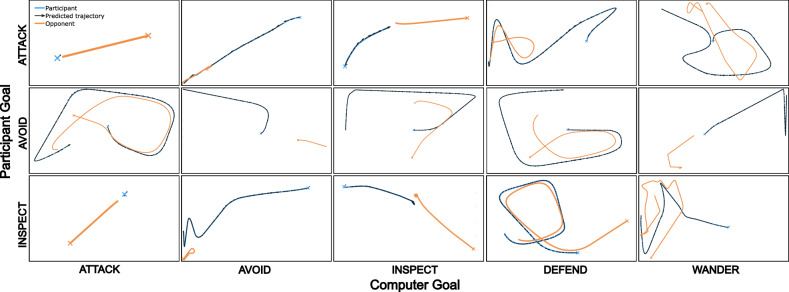
Posterior predictive checks illustrating the overall fit of the model to the data. Example trials from the *Attack*, *Avoid*, and *Inspect* goals are crossed with the five movement profiles of the opponent. The selected trials are those closest to the group’s median mean-squared error, representing a typical degree of model fit. The observed participant’s trajectory is plotted in blue and the observed computer opponent’s trajectory is orange. The model’s predicted trajectories are given by the black arrows originating from the blue line.

To evaluate how well the model can reproduce movement in the task, we used the estimation network’s parameter estimates for each person on each trial to generate a predicted trajectory given the observed locations of the opponent during the trial. We synchronize the position of the participant’s predicted trajectory with their observed trajectory every 4 frames (15 times per second) to avoid longer trials drifting increasingly far from the true trajectory. To visualize the model’s prediction, we display black arrows pointing to the participant’s predicted location overlaid on top of the participant’s actual behavior (blue) alongside the opponent’s behavior (orange). Arrows that track the blue line closely are indicative of good model performance. Arrows that differ in length compared to the blue line (overlap, or do not connect) represent errors in motivational weighting, while arrows that differ in angle compared to the blue line (making it “fuzzy”) represent errors in preferred location. To quantitatively assess model fit, we calculate the mean squared Euclidean distance in pixels (1920 $\times$ 1080p resolution) between the predicted and observed locations of the participant before each resynchronization (see Table 2). We show an example trial from each pairwise combination of *Attack*, *Avoid*, and *Inspect* goals in Fig. 4.

**Table 2:** Posterior prediction error.

					Computer Goal		
			Aggressive	Shy	Curious	Defensive	Wander
Participant Goal	Attack	Mean	109.2	171.5	182.2	162.4	137.7
		Median	41.9	23.3	39.2	38.8	48.2
	Avoid	Mean	155.0	447.4	161.1	134.4	118.9
		Median	54.1	16.8	40.0	43.8	29.1
	Inspect	Mean	111.6	464.5	83.3	105.8	255.3
		Median	49.2	15.5	9.3	36.9	20.2

For each combination of participant and computer goals, we report the mean and median mean-squared error in pixels (1920 $\times$ 1080p resolution) across all trials.

In general, the model provides a good approximation of human behavior. The MSE reported in Table 2 is the average deviation within a 66.67 ms period in squared pixels. Across all goals, there is strong right-skew, as indicated by a much higher group mean than median. These poorly-accounted for outliers, which only represent a small fraction of the data, make comparisons between groups difficult to make with any confidence. An ANOVA on MSE overwhelmingly favors the null model ($BF_{10} = 941.4$). But, by excluding 0.5% of the data on each tail of the distribution to remove extreme outliers (99% retention) the best ANOVA has both the participant’s goal and the computer behavioral profile as predictors ($BF_{10} = 49.3$). This subset will be used for post-hoc comparisons.

The model excels similarly well at reproducing attacking and inspecting behavior ($BF_{10} = 0.02$), while doing slightly worse at avoidance behavior compared to attacking ($BF_{10} = 747.2$) and inspecting ($BF_{10} = 96.3$). Additionally, predictions are better when the computer player is shy compared to when they are aggressive ($BF_{10} > 1000$) or defensive ($BF_{10} = 17.4$). This is no surprise – attacking behavior is relatively simple – the participant only needs to consider local objectives like approaching the opponent (preferred distance near 0) and pace matching to intercept an escape attempt. However, when collisions are aversive, like in the avoid and inspect conditions, the participant must consider their global location in space to avoid being trapped in a corner.

### Classification

With the identifiability and accuracy of the model established, we can finally move on to examining how much (if) it improves inferences about latent motives. This brings us to the focal goal of the paper, which is to test the influence of model parameters in latent motive classification. The aim here was to compare how well we could recover a participant’s latent motive from their behavior, either by directly using their movement during the trial (model-free inference) or by including some model-based information or some summary statistics that deliberately select features for the network to learn. In essence, we are testing whether using a model or statistical summary to extract features from the raw movement data is an effective way to improve classification accuracy on an intent inference / ToM problem.

**Figure 5:**
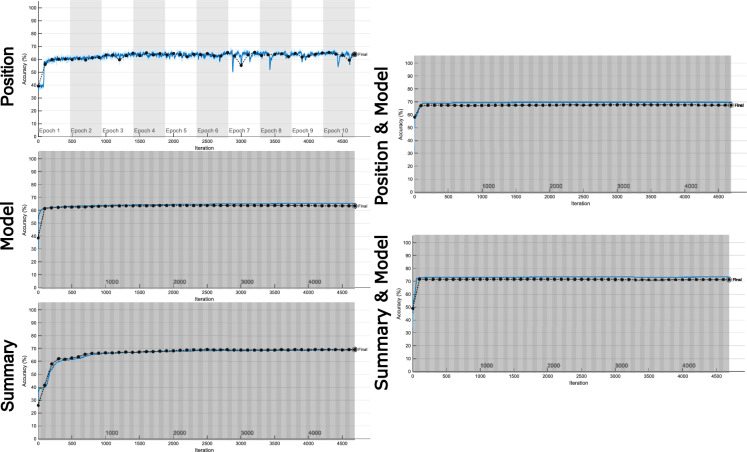
Classification network training plots. Plots show training progress for each classification network. The training accuracy (blue line) is updated each iteration, while validation accuracy (black line) is updated every 100 iterations.

To begin assessing classifier performance, we examine training trajectories for each network, shown in Fig. 5. A clear distinction can be drawn between the position classifier learning from raw multi-feature time-series data and all other classifiers learning from simple feature data. We can see that the gradient of the time-series network is much less smooth, indicated by large variability in accuracy across sequential training iterations. In contrast, feature-based networks all had relatively smooth training trajectories, efficiently reaching a stable minimum.

Model-based features were learned faster than summary statistics, while position was relatively efficient but unstable. For the ensemble models, when the network must simply aggregate predictions from two other networks (Position & Model and Summary & Model), training is exceptionally fast and stable, more so than either network individually.

Put together, scaffolding predictions with model-based inference is substantially more effective than tabula rasa learning or statistical summaries alone. Furthermore, both position (raw) and statistical learning are enhanced by model-based information, making training both faster and more stable. For a task where a neural network has to be (re-)trained, we suspect that complementing predictions with model-based features is likely to be helpful.

#### Classification accuracy

Beyond the rate and stability of model convergence, we can also look at how well the different approaches do in terms of their asymptotic classification performance. Here, we compare both the different types of networks as well as human performance on the classification task outlined above. While humans likely make these judgments based on substantially smaller training sets, this comparison allows us to evaluate how well the classifiers might do at making social inferences about latent motives from observed behavior when they are supplemented with sufficient data.

Classification accuracy for the training, validation, and test data aggregated across all trial lengths is reported in Table 3. All classifiers and human groups showed performance well above chance (33%). All human participants showed similar accuracy between 63.8% and 65.4% correct ($BF_{10} = 0.004$), whether they had monetary incentives (SONA Control compared to Prolific Control), or reported to have been diagnosed with ASD (Prolific Control compared to Prolific ASD), so we combine them into a single real-time comparison group. Of the machine classifiers, the model parameter-only and the position-only classifiers had similarly poor accuracy on the test set ($BF_{10} = 0.07$), slightly above that of human accuracy at 66.0% and 66.7%, respectively. The summary statistics-only classifier performed slightly better, with a test accuracy of 68.3% ($BF_{10} > 1000$) and the addition of model parameters improved classification compared to positions ($BF_{10} > 1000$) or summary statistics alone ($BF_{10} > 1000$), with the best classifier combining summary statistics and model parameters for an accuracy of 72.0%, indicating that cognitive model-based information both stabilized and improved classification. All networks but the position-only classifier showed signs of very mild overfitting, indicated by a test accuracy that was slightly lower than training accuracy. This suggests that networks were sufficiently large and trained sufficiently long to where they had just started to learn information specific to the training set, but nonetheless perform well on held-out data.

To further compare classifier performance, we took the posterior probability assigned to the true class and used it to calculate the log likelihood of each goal given the data. Results, shown in Table 3, echo accuracy comparisons for classifiers receiving one type of data, with the summary statistic classifier performing the best and the position classifier performing slightly worse than the model classifier. On its own, this suggests that carefully selected features, whether they are summary statistics or cognitive model parameters, may be more informative of intent than letting the network self-select features.

A key question of this paper is whether the *addition* of model parameters that are derived from the same data (positions) can improve performance relative to either self-selected features (position classifier) or common statistical features (summary statistic classifier) alone. Here, model-based information substantially improved the performance of both classifiers using position information and classifiers using statistical information – resulting in the Summary and Model classifier performing the best out of all the classifiers we tested. This seems to indicate that careful feature selection, using both statistical and generative models of the data, can be an efficient and effective way to extract latent motives from observed human behavior without requiring the large volume of raw data that participants produce on each trial. Critically, all of the models achieved or exceeded human performance on the task. This suggests that the model alone can classify intent as well as a person can, and that the AI systems we developed here can outperform humans on a social inference task by a substantial margin.

**Table 3:** Human benchmark accuracy from the three samples of online participants with training accuracy, validation accuracy, test accuracy, and the log likelihood of predicting the correct test class for each classifier input for *Attack*, *Avoid*, and *Inspect* goals.

Classifier inputs	Training	Validation	Test	LL
SONA control	–	–	63.8%	–
Prolific control	–	–	65.4%	–
Prolific ASD	–	–	63.9%	–
Model	66.3%	63.4%	66.0%	− 49,733
Position	64.3 %	63.9%	66.7%	− 49,771
Position and model	70.0%	67.6%	68.9%	− 45,753
Summary	69.8%	69.4%	68.3%	− 49,608
Summary and model	73.7%	71.4%	72.0%	− 44,304

#### Real-time classification

Thus far, we have explored goal classification aggregated across trials of different lengths. Because all networks were trained to estimate parameters from or classify data of varying lengths through purposefully censoring, or cutting off, the end of each trial, these networks can be applied iteratively during a trial. By continuously feeding in input data and extracting posterior probabilities, these networks can perform real-time intent inference. In other words, with each additional unit of time, networks can update their estimates of model parameters or goal probabilities. To our knowledge, generative cognitive models have never been fit in real time, which is necessary to have cognitive models augment real-time goal inference. When exploring parameter recovery, we saw that most local parameters could be well-fit by four or fewer seconds of observed data, but that global parameters saw some recovery benefits from an additional four to seven seconds of information, further constraining the most likely parameter values. Thus, we might expect goal classification accuracy to improve as observable trial duration increases.

To test this, we took each trial of data and fed it into the neural network at the sampling rate (60 Hz) to extract posterior probabilities for each trial and time point. The network performance was fast enough that it took less than 17 ms for each prediction, meaning it could be applied iteratively faster than the computer monitor could be refreshed – allowing for fully real-time inference.

**Figure 6:**
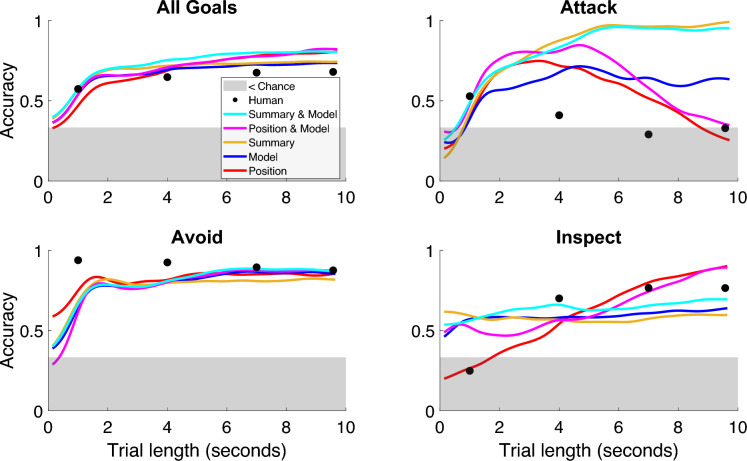
Real-time classification accuracy. Each plot shows a kernel smoothing regression of binary goal-classification accuracy from machine classifiers across different lengths of trials in seconds. Additionally, human accuracy is aggregated across individuals and plotted according to the 1-s, 4-s, 7-s, and 10-s conditions. The top left plot shows aggregate performance across all goals for each classifier and human, while the remaining plots separate performance by the goal being inferred.

Real-time goal classification accuracy is displayed for each of the three goals and in aggregate in Fig. 6. While binary classification accuracy obscures some uncertainty regarding the probability that a network assigns to the most likely class, it allows for direct comparisons with human performance at the four elicited time points (black dots). In the aggregate (top-left panel), there does not appear to be a substantial difference between human and machine performance, as both show steadily increasing accuracy over the course of a trial.

However, humans and machines diverge quite sharply in their performance on specific conditions. Looking at specific goals, we see that machines greatly outperform humans when inferring the Attack goal. The decrease in accuracy from both humans and (some) machines when classifying long Attack trials may be due to the best attackers (those who swiftly collide with their opponent) producing shorter trials; thus, long trials are only produced by attackers who are failing to collide with their target. The inclusion of summary statistics appears to prevent this decrease in accuracy. Likewise, machines also tend to outperform humans at the beginning of an Inspect trial, but humans seem to be on-par if not better at inferring an Inspect goal later in the trial. This may be because the first second of an Attack and Inspect trial often appear very similar to a person (move towards the opponent), but a machine might pick up on minute differences regarding changes in acceleration (i.e., jerk) that help to differentiate between attacking and inspecting. But as the trial evolves, humans seem to be better at delineating who is inspecting and who is attempting to attack despite failing to make contact. However, position data appears to finally catch up and surpass human inferences with enough time. Lastly, humans appear to be far superior when inferring an Avoid trial after only one second of behavior, but ultimately machines catch up as the trial evolves, showing strong classification accuracy after approximately six seconds of observation.

Comparing the machine-based classifiers that received one type of input data, we see a pattern where the position-only network has extremely high accuracy for Attack goals early in a trial, but accuracy below chance later in the trial, with the opposite pattern observed for Inspect goals. This may suggest that the position-only network cannot reliably distinguish between a failed attacker and an inspector, instead over-relying on trial duration to determine class assignment, even when trained on censored data. Focusing on how model parameters can augment real-time inference, we see the ensemble models typically having accuracy similar to or higher than either component model across the different trial lengths. This suggests that the model parameters capture unique intent-relevant information that neural networks can effectively integrate with information derived from raw positions or summary statistics in real time.

## Discussion

This study demonstrated not only the possibility but the potentially substantial benefit of using real-time cognitive models to make inferences about latent motives. Results suggest that real-time cognitive models can meaningfully overcome key barriers to machine ToM by improving AI systems’ ability to infer latent motives. By all metrics – training speed, robustness / stability, and asymptotic performance – using informative features extracted from a cognitive model improved classification relative to networks trained on raw data or statistical summaries. The best-performing approach integrated both researcher-chosen features and model-based features, indicating that human-informed information pre-processing can help with filtering and structuring data for intent inference. While the raw data about ship positions was only marginally worse, there appears to be no advantage to allowing the neural network to select its own features from the raw data. This makes some sense – humans (modelers, researchers) have some meta-theoretical knowledge about what aspects of behavior are indicators of intent, making it possible to combine human judgments about feature importance with the automatic feature extraction that deep neural networks perform.

Critically, the AI systems we developed here match or exceed human intent inference in this experimental task, despite humans being stereotypically more adept at extracting social motives. This indicates that stumbling blocks for socially-intelligent AI may be overcome with effective feature selection – making inferences using generative cognitive models, deriving informative summaries from behavioral data, and allowing deep neural networks to carry out automated feature selection. In some cases, the benefit from using multiple approaches may be marginal; yet in many real-world applications, marginal improvements in AI can have important consequences. Even marginal improvements in our ability to infer the intent of another car to be passing versus merging, for example, can make a difference in the health and safety of the cars’ occupants as well as millions of dollars in damages. However we must first assess the degree to which cognitive model parameters improve real-world AI systems engaged in complex tasks like driving and how best to utilize that information (e.g., when should a semi-autonomous vehicle automatically adjust trajectory and when should it instead notify a human driver).

Beyond pure performance, cognitive models also confer benefits in terms of transparency. An AI that understands *why* a person behaves in a certain way, and can refer to meaningful parameters that correspond to real cognitive or neural processes, is potentially both more robust and trustworthy. This may help build trust between AI and its users and facilitate cooperation based on goals and motivations rather than past behavior. Thus, cognitive models might be an alternative approach to developing better explainable AI (XAI)^67,68^.

XAI, or an AI system that can be understood by a human, can be accomplished in a multitude of ways. First, some models are simple enough to be understood at an algorithmic level, like decision trees^9^ or rule-based models^7^. These are typically referred to as transparent or white-box models. When models are not directly interpretable (black-box), the system might be augmented with some form of ad-hoc explainer that aims to describe why a system made a prediction, which can foster a local understanding specific to that instance^9^. A global understanding of how the model operates can be improved through tools like model visualization^69^. Cognitive models might improve the explainability of an AI system by highlighting interpretable model parameters (i.e., features) that had a strong influence on a judgment or inference. Furthermore, if cognitive models can help an AI system exhibit more human-like behavior, a person might be able to build a better mental model of the system which could improve trust^70,71^.

To realize these benefits in the real world, a number of limitations must be overcome. First, the present work only selects among three different known goals. In the real world, AI systems might contend with many more goals, including goals that are unknown^72^. This might require larger networks with more extensive training, as well as anomaly detection and novel category generation approaches. Even when the set of goals is incomplete, these cognitive model parameters can define a parameter space over which novel category inferences are made. Future work might aim to simultaneously classify the intent of multiple participants with a wider variety of goals to pursue. Second, the poor identifiability of movement variability and moderate recovery of spatial sensitivity and spatial strength limit their interpretability, and therefore the degree to which they help to make input data explainable for a human and useful for an AI system. Parameter estimation networks might be improved through hierarchical estimation and by optimizing posterior prediction error instead of parameter estimation error. Lastly, further work is necessary to determine the generalizability of the present findings. Results need to be replicated among many more people as the present human benchmark only made inferences about the latent motives of eight different individuals. This also includes validating different cognitive models for a variety of tasks. The present task, while quite complex in terms of temporal and spatial continuity compared to many ToM-related tasks^43^, is still tightly constrained, with only one modality of behavior (movement), and is far from the complexity of three-dimensional real-world environments. Additionally, classification of latent motives (i.e., desires) is only one of many abilities that might be considered part of ToM – identifying new ToM tasks, appropriate models, and how to generalize inferences across these tasks will be key to equipping machines with effective theory of mind.

Despite the overall superiority of machine-based classifiers, comparing human and machine real-time inference for each goal revealed some striking differences where machines are better at classifying attackers, while humans show some advantages when it comes to early detection of avoidance. This may reflect an interpretation bias, where ambiguous movement might be more easily interpreted as avoidance behavior^73^. It is also possible that human judges are influenced by a correspondence bias^74,75^ where situational constraints are not given enough relative consideration. This could lead to a person judging an attacker who is repeatedly missing their target as an inspector, perhaps due to unrealistic expectations about how easy it is to control the ship, making it appear that misses are purposeful^75^. These key differences between machine-based and human intent inference might be leveraged in the composite decision of a human operator with an AI assistant^76,77^.

More broadly, cognitive modeling may serve as a key tool among a wider toolbox of methods available when constructing AI. Fundamentally, attributing behavior to some latent motives, abilities, or cognitive capacities allows people to function in a social world. Equipping AI with these capacities may make it safer, more effective, and more human (in positive ways, as opposed to biased ways), pending validation in real-world contexts. The additional predictive power gleaned from cognitive model parameters will surely depend on the task and the model used to approximate the task, but the explanatory power (*why* is a person acting this way?) cannot be matched by any other approach. As always, future work needs to be done to identify what types of tasks and models yield the most benefit from these model-based insights – but the provenance of even a new model like the one presented here signal that it is a promising avenue for developing safer and more effective AI.

## Data Availability

All data, materials, and code are available on https://osf.io/cftuy/.

## References

[1] Sally, D. & Hill, E. The development of interpersonal strategy: Autism, theory-of-mind, cooperation and fairness. *J. Econ. Psychol.* **27**, 73–97 (2006).

[2] Heider, F. & Simmel, M. An experimental study of apparent behavior. *Am. J. Psychol.* **57**, 243–259 (1944).

[3] Premack, D. & Woodruff, G. Does the chimpanzee have a theory of mind?. *Behav. Brain Sci.* **1**, 515–526 (1978).

[4] Sanfey, A. G. Social decision-making: insights from game theory and neuroscience. *Science* **318**, 598–602 (2007). 17962552 10.1126/science.1142996

[5] Rusch, T., Steixner-Kumar, S., Doshi, P., Spezio, M. & Gläscher, J. Theory of mind and decision science: Towards a typology of tasks and computational models. *Neuropsychologia* **146**, 107488 (2020). 32407906 10.1016/j.neuropsychologia.2020.107488

[6] Von Eschenbach, W. J. Transparency and the black box problem: Why we do not trust ai. *Philos. Technol.* **34**, 1607–1622 (2021).

[7] Hassija, V. et al. Interpreting black-box models: a review on explainable artificial intelligence. *Cogn. Comput.* **16**, 45–74 (2024).

[8] Nourani, M., Kabir, S., Mohseni, S. & Ragan, E. D. The effects of meaningful and meaningless explanations on trust and perceived system accuracy in intelligent systems. *In Proceedings of the AAAI Conference on Human Computation and Crowdsourcing* **7**, 97–105 (2019).

[9] Mohseni, S., Zarei, N. & Ragan, E. D. A multidisciplinary survey and framework for design and evaluation of explainable ai systems. *ACM Trans. Interact. Intell. Syst. (TiiS)* **11**, 1–45 (2021).

[10] Kadar, E. E. Mind the gap: a theory is needed to bridge the gap between the human skills and self-driving cars. *Robot. Well-Being* 55–65 (2019).

[11] Chen, S., Zhang, S., Shang, J., Chen, B. & Zheng, N. Brain-inspired cognitive model with attention for self-driving cars. *IEEE Trans. Cogn. Dev. Syst.* **11**, 13–25 (2017).

[12] Busemeyer, J. R. & Diederich, A. *Cognitive modeling* (Sage, 2010).

[13] Farrell, S. & Lewandowsky, S. *Computational modeling of cognition and behavior* (Cambridge University Press, 2018).

[14] Wang, Z. J. & Busemeyer, J. R. *Cognitive choice modeling* (MIT Press, 2021).

[15] Green, D. M. & Swets, J. A. Signal detection theory and psychophysics. *Psychol. Bull.* **75**, 424–429. 10.1901/jeab.1969.12-475 (1966).

[16] Busemeyer, J. R., Gluth, S., Rieskamp, J. & Turner, B. M. Cognitive and neural bases of multi-attribute, multi-alternative, value-based decisions. *Trends Cogn. Sci.* **23**, 251–263 (2019). 30630672 10.1016/j.tics.2018.12.003

[17] Mulder, M., Van Maanen, L. & Forstmann, B. Perceptual decision neurosciences-a model-based review. *Neuroscience* **277**, 872–884 (2014). 25080159 10.1016/j.neuroscience.2014.07.031

[18] Ratcliff, R. A theory of memory retrieval. *Psychol. Rev.* **85**, 59–108. 10.1037/0033-295X.85.2.59 (1978).

[19] Cuzzolin, F., Morelli, A., Cirstea, B. & Sahakian, B. J. Knowing me, knowing you: theory of mind in ai. *Psychol. Med.* **50**, 1057–1061 (2020). 32375908 10.1017/S0033291720000835 PMC7253617

[20] Schossau, J. & Hintze, A. Towards a theory of mind for artificial intelligence agents. In *Artificial Life Conference Proceedings 35*, vol. 2023, 21 (MIT Press One Rogers Street, Cambridge, MA 02142-1209, USA journals-info, 2023).

[21] McIlroy-Young, R., Sen, S., Kleinberg, J. & Anderson, A. Aligning superhuman ai with human behavior: Chess as a model system. In *Proceedings of the 26th ACM SIGKDD International Conference on Knowledge Discovery & Data Mining*, 1677–1687 (2020).

[22] Campbell, M., Hoane, A. J. Jr. & Hsu, F.-H. Deep blue.. *Artif. Intell.* **134**, 57–83 (2002).

[23] Bowling, M., Burch, N., Johanson, M. & Tammelin, O. Heads-up limit hold’em poker is solved. *Science* **347**, 145–149 (2015). 25574016 10.1126/science.1259433

[24] Brown, N. & Sandholm, T. Superhuman ai for multiplayer poker. *Science* **365**, 885–890 (2019). 31296650 10.1126/science.aay2400

[25] Chen, X., Deng, X. & Teng, S.-H. Settling the complexity of computing two-player nash equilibria. *J. ACM (JACM)* **56**, 1–57 (2009).

[26] Silver, D. et al. Mastering the game of go without human knowledge. *Nature* **550**, 354–359 (2017). 10.1038/nature24270 29052630

[27] Silver, D. et al. Mastering the game of go with deep neural networks and tree search. *Nature* **529**, 484–489 (2016). 10.1038/nature16961 26819042

[28] Baron-Cohen, S., Leslie, A. M. & Frith, U. Does the autistic child have a “theory of mind’’?. *Cognition* **21**, 37–46 (1985). 2934210 10.1016/0010-0277(85)90022-8

[29] Rabinowitz, N. et al. Machine theory of mind. In *International conference on machine learning*, 4218–4227 (PMLR, 2018).

[30] Narang, S., Best, A. & Manocha, D. Inferring user intent using bayesian theory of mind in shared avatar-agent virtual environments. *IEEE Trans. Visual Comput. Graphics* **25**, 2113–2122 (2019). 10.1109/TVCG.2019.2898800 30762558

[31] Kosinski, M. Evaluating large language models in theory of mind tasks. *Proc. Natl. Acad. Sci.* **121**, e2405460121 (2024). 39471222 10.1073/pnas.2405460121 PMC11551352

[32] Xu, H., Zhao, R., Zhu, L., Du, J. & He, Y. Opentom: A comprehensive benchmark for evaluating theory-of-mind reasoning capabilities of large language models. *arXiv preprint* arXiv:2402.06044 (2024).

[33] Lu, Y., Aleta, A., Du, C., Shi, L. & Moreno, Y. Llms and generative agent-based models for complex systems research. *Phys. Life Rev.* (2024). 10.1016/j.plrev.2024.10.013 39486377

[34] Nguyen, T. N. & Gonzalez, C. Theory of mind from observation in cognitive models and humans. *Top. Cogn. Sci.* **14**, 665–686 (2022). 34165919 10.1111/tops.12553

[35] Mao, Y., Liu, S., Ni, Q., Lin, X. & He, L. A review on machine theory of mind. *IEEE Transactions on Computational Social Systems* (2024).

[36] Doshi, A. & Trivedi, M. M. Tactical driver behavior prediction and intent inference: A review. In *2011 14th International IEEE Conference on Intelligent Transportation Systems (ITSC)*, 1892–1897 (IEEE, 2011).

[37] Yepes, J. L., Hwang, I. & Rotea, M. New algorithms for aircraft intent inference and trajectory prediction. *J. Guid. Control. Dyn.* **30**, 370–382 (2007).

[38] Patel, J. et al. Give us a hand, mate! a holistic review of research on human-machine teaming. *BMJ Mil Health* (2024). 10.1136/military-2024-002737 PMC12505082 39719381

[39] Lyn Paul, C., Blaha, L. M., Fallon, C. K., Gonzalez, C. & Gutzwiller, R. S. Opportunities and challenges for human-machine teaming in cybersecurity operations. In *Proceedings of the human factors and ergonomics society annual meeting*, 63, 442–446 (SAGE Publications Sage CA: Los Angeles, CA, 2019).

[40] Fang, J., Schooler, L. & Shenghua, L. Machine learning strategy identification: A paradigm to uncover decision strategies with high fidelity. *Behav. Res. Methods* 1–22 (2022). 10.3758/s13428-022-01828-1 35378675

[41] Vaswani, A. *et al.* Attention is all you need. *Adv. Neural Inf. Process. Syst.* **30** (2017).

[42] Liefooghe, B. & van Maanen, L. Three levels at which the user’s cognition can be represented in artificial intelligence. *Front. Artif. Intell.* **5**, 293 (2023). 10.3389/frai.2022.1092053 PMC9880274 36714204

[43] Aru, J., Labash, A., Corcoll, O. & Vicente, R. Mind the gap: Challenges of deep learning approaches to theory of mind. *Artif. Intell. Rev.* **56**, 9141–9156 (2023).

[44] Labash, A., Aru, J., Matiisen, T., Tampuu, A. & Vicente, R. Perspective taking in deep reinforcement learning agents. *Front. Comput. Neurosci.* **14**, 69 (2020). 32792931 10.3389/fncom.2020.00069 PMC7390927

[45] Ratcliff, R. & Strayer, D. Modeling simple driving tasks with a one-boundary diffusion model. *Psychon. Bull. Rev.* **21**, 577–589 (2014). 24297620 10.3758/s13423-013-0541-x PMC4032619

[46] Liu, P., Zhao, J., Zhang, F. & Yeo, H. Modeling decision-making process of drivers during yellow signal phase at intersections based on drift-diffusion model. *Transport. Res. F: Traffic Psychol. Behav.* **105**, 368–384 (2024).

[47] Yoo, S. B. M., Hayden, B. Y. & Pearson, J. M. Continuous decisions. *Philos. Trans. R. Soc. B* **376**, 20190664 (2021). 10.1098/rstb.2019.0664 PMC7815426 33423634

[48] Rasanan, A. H. H. et al. Beyond discrete-choice options. *Trends Cogn. Sci.* (2024). 10.1016/j.tics.2024.07.004 39138030

[49] Turner, B. M. & Sederberg, P. B. Approximate Bayesian computation with differential evolution. *J. Math. Psychol.* **56**, 375–385 (2012).

[50] Kvam, P. D. & Turner, B. M. Reconciling similarity across models of continuous selections. *Psychol. Rev.* **128**, 766–786 (2021). 34081510 10.1037/rev0000296

[51] Kvam, P. D., Sokratous, K., Fitch, A. K. & Vassileva, J. Comparing likelihood-based and likelihood-free approaches to fitting and comparing models of intertemporal choice. *PsyArXiv*10.31234/osf.io/gqzse (2025). 10.3758/s13428-025-02779-z PMC12339600 40790370

[52] Radev, S. T., Mertens, U. K., Voss, A., Ardizzone, L. & Köthe, U. Bayesflow: Learning complex stochastic models with invertible neural networks (2020). arXiv:2003.06281. 10.1109/TNNLS.2020.3042395 33338021

[53] Sokratous, K., Fitch, A. & Kvam, P. D. How to ask twenty questions and win: Machine learning tools for assessing preferences from small samples of willingness-to-pay prices. *J. Choice Model.* **48**, 100418 (2023).

[54] Cranmer, K., Brehmer, J. & Louppe, G. The frontier of simulation-based inference. *Proc. Natl. Acad. Sci.* **117**, 30055–30062 (2020). 32471948 10.1073/pnas.1912789117 PMC7720103

[55] Shiffrin, R. M. Is it reasonable to study decision-making quantitatively?. *Top. Cogn. Sci.* **14**, 621–633 (2022). 34050714 10.1111/tops.12541

[56] Kleinberg, J., Ludwig, J., Mullainathan, S. & Raghavan, M. The inversion problem why algorithms should infer mental state and not just predict behavior. *Perspect. Psychol. Sci.* 17456916231212138 (2023). 10.1177/17456916231212138 38085919

[57] Rosenthal, I. A., Hutcherson, C. A., Adolphs, R. & Stanley, D. A. Deconstructing theory-of-mind impairment in high-functioning adults with autism. *Curr. Biol.* **29**, 513–519 (2019). 30686740 10.1016/j.cub.2018.12.039

[58] Langley, C., Cirstea, B. I., Cuzzolin, F. & Sahakian, B. J. Theory of mind and preference learning at the interface of cognitive science, neuroscience, and ai: A review. *Front. Artif. Intell.* **5**, 778852 (2022). 35493614 10.3389/frai.2022.778852 PMC9038841

[59] Murray, A., Mannion, A., Chen, J. L. & Leader, G. Gaming disorder in adults with autism spectrum disorder. *J. Autism Dev. Disord.* 1–8 (2022). 10.1007/s10803-021-05138-x PMC9114087 34184139

[60] Lewin, K. *A dynamic theory of personality* (McGraw-Hill, 1935).

[61] Townsend, J. T. & Busemeyer, J. R. Approach-avoidance: Return to dynamic decision behavior. In *Cognitive Processes the Tulane Flowerree Symposia on Cognition*, 107 (2014).

[62] Hamlin, R. P. “the gaze heuristic:’’ biography of an adaptively rational decision process. *Top. Cogn. Sci.* **9**, 264–288 (2017). 28220988 10.1111/tops.12253

[63] Kingma, D. P. & Ba, J. Adam: A method for stochastic optimization (2014).

[64] Gal, Y. & Ghahramani, Z. Dropout as a bayesian approximation: Representing model uncertainty in deep learning. In *International conference on machine learning*, 1050–1059 (PMLR, 2016).

[65] Xu, K. et al. How neural networks extrapolate: From feedforward to graph neural networks. *arXiv preprint* arXiv:2009.11848 (2020).

[66] Steingroever, H., Wabersich, D. & Wagenmakers, E.-J. Modeling across-trial variability in the wald drift rate parameter. *Behav. Res. Methods* 1–17 (2020). 10.3758/s13428-020-01448-7 PMC8219596 32948979

[67] Miller, T. Explanation in artificial intelligence: Insights from the social sciences. *Artif. Intell.* **267**, 1–38 (2019).

[68] Taylor, J. E. T. & Taylor, G. W. Artificial cognition: How experimental psychology can help generate explainable artificial intelligence. *Psychon. Bull. Rev.* **28**, 454–475 (2021). 33159244 10.3758/s13423-020-01825-5

[69] Liu, M. et al. Towards better analysis of deep convolutional neural networks. *IEEE Trans. Visual Comput. Graphics* **23**, 91–100 (2016). 10.1109/TVCG.2016.2598831 27576252

[70] Nourani, M. et al. Anchoring bias affects mental model formation and user reliance in explainable ai systems. In *Proceedings of the 26th International Conference on Intelligent User Interfaces*, 340–350 (2021).

[71] Steyvers, M. & Kumar, A. Three challenges for ai-assisted decision-making. *Perspect. Psychol. Sci.* **19**, 722–734 (2024). 37439761 10.1177/17456916231181102 PMC11373149

[72] Han, K., Rebuffi, S.-A., Ehrhardt, S., Vedaldi, A. & Zisserman, A. Autonovel: Automatically discovering and learning novel visual categories. *IEEE Trans. Pattern Anal. Mach. Intell.* **44**, 6767–6781 (2021). 10.1109/TPAMI.2021.3091944 34166184

[73] Hirsch, C. R., Clark, D. M. & Mathews, A. Imagery and interpretations in social phobia: Support for the combined cognitive biases hypothesis. *Behav. Ther.* **37**, 223–236 (2006). 16942974 10.1016/j.beth.2006.02.001

[74] Jones, E. E. & Harris, V. A. The attribution of attitudes. *J. Exp. Soc. Psychol.* **3**, 1–24 (1967).

[75] Gilbert, D. T. & Malone, P. S. The correspondence bias. *Psychol. Bull.* **117**, 21 (1995). 7870861 10.1037/0033-2909.117.1.21

[76] Hasan, E., Duhaime, E. & Trueblood, J. S. Boosting wisdom of the crowd for medical image annotation using training performance and task features. *Cogn. Res. Principles Implications* **9**, 31 (2024). 10.1186/s41235-024-00558-6 PMC11102897 38763994

[77] Wang, D. et al. From human-human collaboration to human-ai collaboration: Designing ai systems that can work together with people. In *Extended abstracts of the 2020 CHI conference on human factors in computing systems*, 1–6 (2020).

